# Supplementary material for: Glia-neuron coupling via a bipartite sialylation pathway promotes neural transmission and stress tolerance in Drosophila
Source: eLife. 2023 Mar 22;12:e78280. doi: 10.7554/eLife.78280 (PMC10110239; doi:10.7554/eLife.78280)
Supplement: Figure 5—source data 3. — n, number of replicates. ND, not detected. Graphical representation and description of structures are according to the glycan nomenclature (Aoki et al., 2007; Varki et al., 2015b). Accession numbers are from GlyTouCan, the International Glycan Structure Repository (https://glytoucan.org/). All raw mass spectrometric data were deposited at GlycoPost (Watanabe et al., 2021), accession # GPST000260. [file elife-78280-fig5-data3.docx]

| **Glycan #** | **Graphical Representation** | **Description** | **GlyTouCan Accession**  **number** | ***wildtype*** | ***CSAS^-/-^*** | ***DSiaT^-/-^*** |
| --- | --- | --- | --- | --- | --- | --- |
|  |  |  |  | % total profile mean ± sem (n=5) | % total profile mean ± sem (n=3) | % total profile mean ± sem (n=2) |
| **High mannose type** | | | | | | |
| 1 |  | M4N2 | G09724ZC | 1.67 ± 0.35 | 2.26 ± 1.33 | 2.06 ± 1.10 |
| 2 |  | M5N2 | G55220VL | 7.88 ± 1.25 | 7.41 ± 1.26 | 4.20 ± 0.89 |
| 3 |  | M6N2 | G80966KZ | 7.42 ± 1.23 | 6.63 ± 2.70 | 7.35 ± 1.85 |
| 4 |  | M7N2 | G83161QT | 7.47 ± 1.35 | 4.68 ± 2.49 | 9.44 ± 3.33 |
| 5 |  | M8N2 | G14548ZL | 6.58 ± 1.37 | 4.33 ± 2.10 | 8.18 ± 4.19 |
| 6 |  | M9N2 | G60230HH | 8.77 ± 1.69 | 5.54 ± 2.44 | 9.14 ± 6.09 |
| 7 |  | GlcM9N2 | G19958IL | 1.31 ± 0.30 | 0.94 ± 0.33 | 1.49 ± 1.02 |
| **Pauci mannose type** | | | | | | |
| 8 |  | M2N2 | G13008EW | 2.80 ± 0.39 | 3.61 ± 0.30 | 2.27 ± 0.09 |
| 9 |  | M3N2 | G22768VO | 11.09 ± 1.01 | 14.03 ± 3.27 | 8.28 ± 0.19 |
| 10 |  | M2N2F | G42466VF | 8.41 ± 0.36 | 12.14 ± 1.72 | 6.74 ± 0.53 |
| 11 |  | M3N2F | G82348BZ | 29.14 ± 3.93 | 32.92 ± 5.15 | 31.16 ± 13.55 |
| **Complex type** | | | | | | |
| 12 |  | NM3N2 | G61751GZ | 1.22 ± 0.26 | 1.79 ± 0.23 | 1.70 ± 1.09 |
| 13 |  | NM3N2F | G07483YN | 4.33 ± 1.25 | 1.19 ± 0.26 | 3.91 ± 1.47 |
| 14 |  | N2M3N2F | G80858MF | 1.39 ± 0.74 | 2.28 ± 0.52 | 2.61 ± 1.30 |
| 15 |  | N3M3N2F | G61207RZ | 0.32 ± 0.05 | 0.25 ± 0.10 | 1.47 ± 0.21 |
| **Sialylated glycans** | | | | | | |
| 16 |  | SAGalNM3N2 | G06209KS | 0.07 ± 0.04 | ND | ND |
| 17 |  | SAGalN2M3N2 | G04791QM | 0.10 ± 0.07 | ND | ND |
