# Supplementary figures and images for: Glia-neuron coupling via a bipartite sialylation pathway promotes neural transmission and stress tolerance in Drosophila
Source: eLife. 2023 Mar 22;12:e78280. doi: 10.7554/eLife.78280 (PMC10110239; doi:10.7554/eLife.78280)

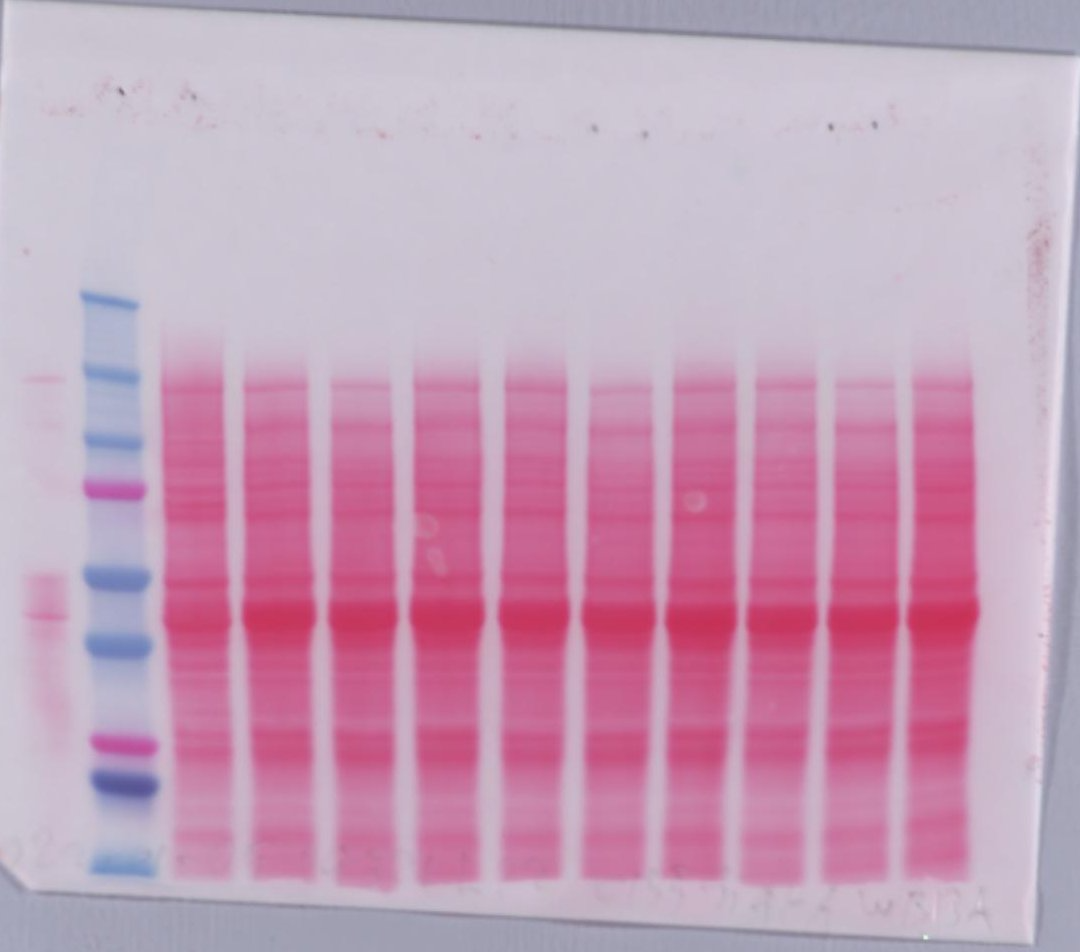

Supplement: Figure 9—source data 2. [file elife-78280-fig9-data2.zip › Figure 9B - Source data R1/PG321L22c155Sia APoS 2022.01.06_10.54.44_Co.tiff]

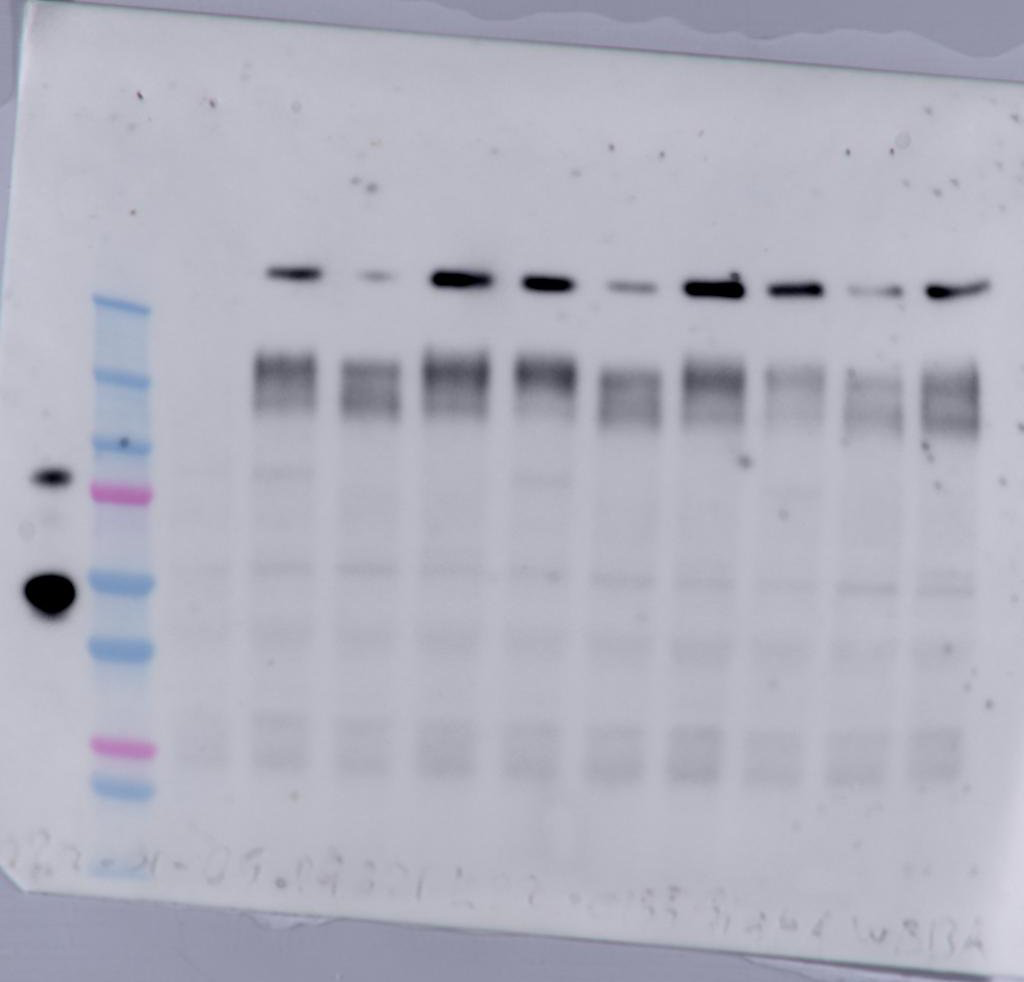

Supplement: Figure 9—source data 2. [file elife-78280-fig9-data2.zip › Figure 9B - Source data R1/PG321L22c155Sia A90s 2022.01.06_19.17.53_Ch+Marker.tif]

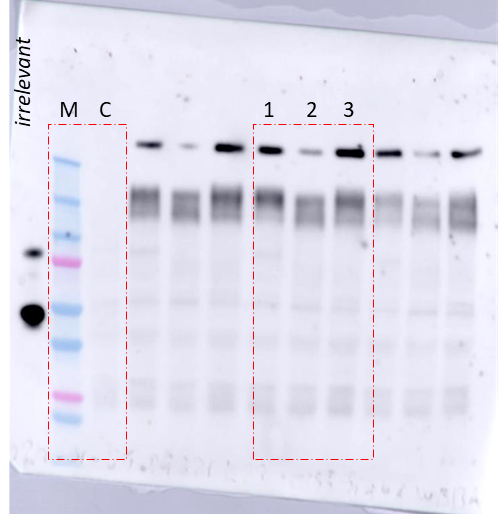


Para-GFP


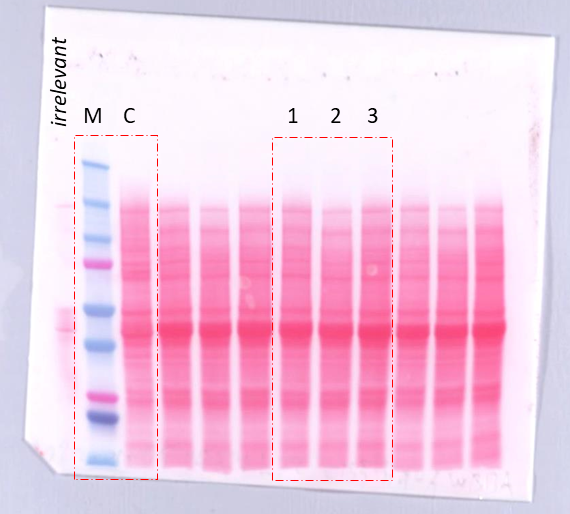

Supplement: Figure 9—source data 2. [file elife-78280-fig9-data2.zip › Figure 9B - Source data R1/Figure 9B - Source data - Labeled R1.docx]

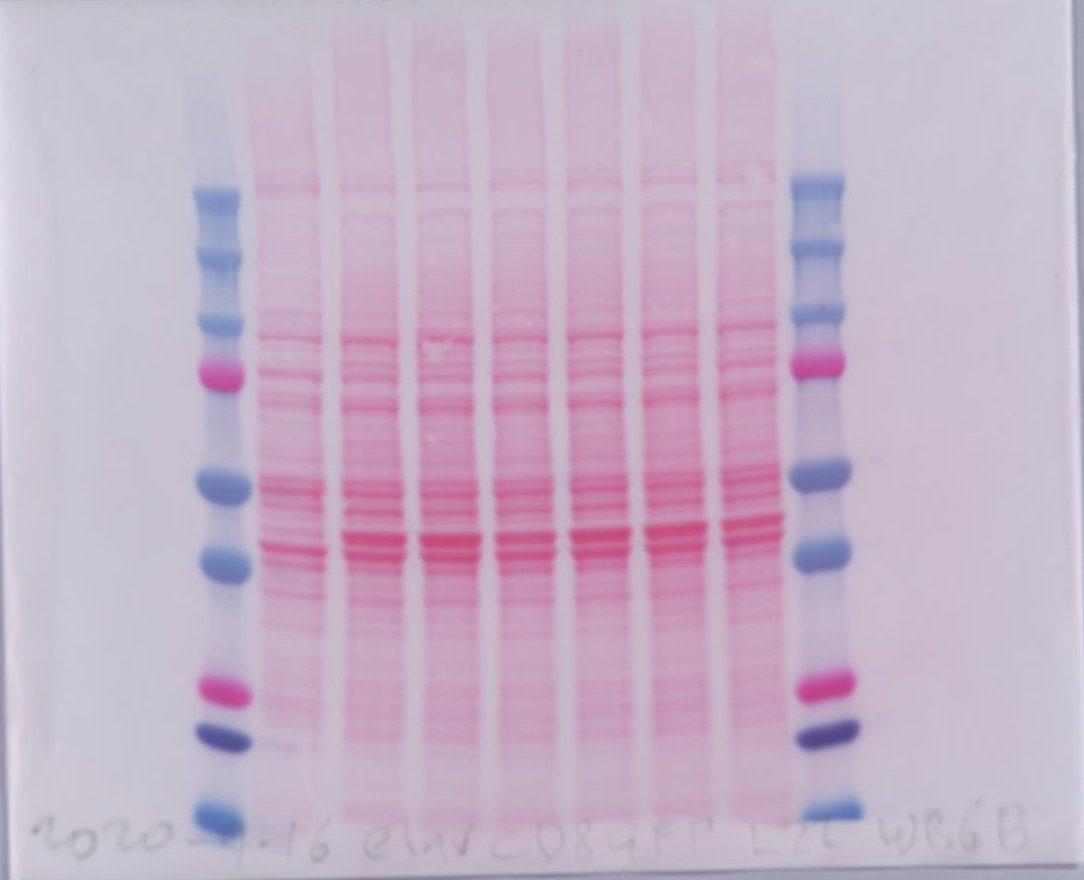

Supplement: Figure 9—figure supplement 1—source data 3. [file elife-78280-fig9-figsupp1-data3.zip › Figure 9 ΓÇôfigure supplement 1C ΓÇô Source data R1/elavCD8gfpL22wb6BPoS 2021.09.17_09.22.25_Co.tiff]

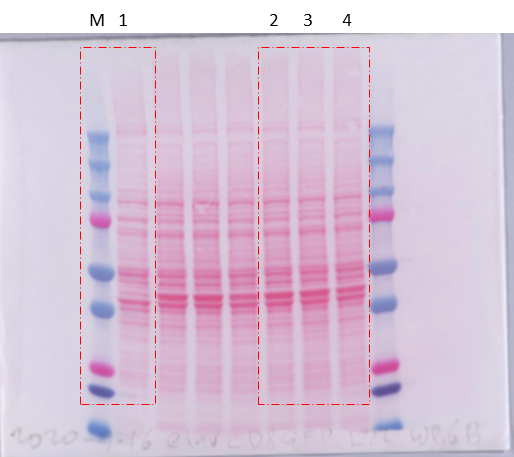

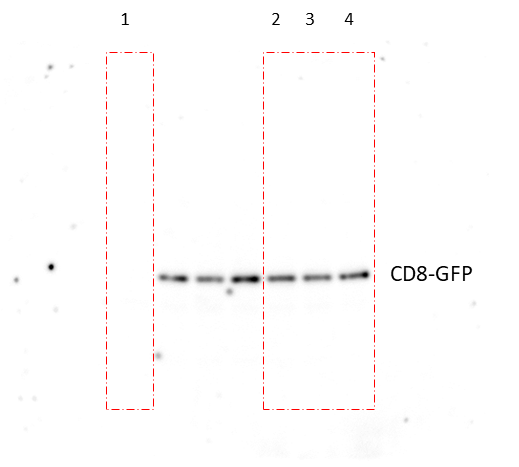

Supplement: Figure 9—figure supplement 1—source data 3. [file elife-78280-fig9-figsupp1-data3.zip › Figure 9 ΓÇôfigure supplement 1C ΓÇô Source data R1/Figure 9 -figure supplement 1C Source data - Labeled R1.docx]
