# Supplementary material for: Glia-neuron coupling via a bipartite sialylation pathway promotes neural transmission and stress tolerance in Drosophila
Source: eLife. 2023 Mar 22;12:e78280. doi: 10.7554/eLife.78280 (PMC10110239; doi:10.7554/eLife.78280)
Supplement: Supplementary file 1. [file elife-78280-supp1.docx]

Supplementary table of genetic strains and transgenic constructs used in the study. Genetic strains created in this study are available per request

| **Genotype/ allele abbreviation** | **Genotype details** | **Source / Reference** |
| --- | --- | --- |
| *WT*  *C155-Gal4*  *repo-Gal4*  *1407-Gal4*  *Act-Gal4*  *C164-Gal4*  *AyGal4*  *UAS-DSiaT-RNAi*  *LexAop2-mCD8-GFP*  *LexAop-GFP.nls*  *LexAop-FLP*  *C155>CD8-GFP*  *R85G01-Gal4*  *R56F03-Gal4*  *UAS-CSAS-RNAi*  *dEAAT1-Gal4*  *Gli-Gal4*  *MZ709-Gal4*  *Mj85b-Gal4*  *UAS-CSAS*  *UAS-CSAS-FLAG*  *UAS-DSiaT*  *para-GFP*  *CSAS^21^*  *CSAS^Mi^*  *DSiaT^L22^*  *DSiaT^S23^*  *BAC-CSAS*  *CSAS-LexA*  *DSiaT-HA*  *UAS-NANS*  *_ _*    *Genotypes generated in crosses*  *CSAS> mCD8-GFP*  *CSAS> GFPnls*  *BAC-CSAS CSAS^21/21^*  *CSAS>FLP _>CSAS CSAS^Mi/Mi^*  *CSAS>_ AyGal4>CSAS*  *CSAS^Mi/Mi^*  *CSAS>FLP AyGal4>CSAS*  *CSAS^Mi/Mi^*  *CSAS^Mi/21^*  *CSAS^21/21^*  *Repo>CSAS CSAS^21/21^*  *Gli>CSAS CSAS^21/21^*  *Mz709>CSAS CSAS^Mi/21^*  *R85G01>CSAS CSAS^Mi/21^*  *R56F03>CSAS CSAS^Mi/21^*  *dEAAT1>CSAS CSAS^Mi/21^*  *C155>CSAS CSAS^21/21^*  *Mj85b>CSAS CSAS^21/21^*  *1407>CSAS CSAS^21/21^*  *_>CSAS CSAS^Mi/21^*  *C155>_ CSAS^Mi/21^*  *Repo>_ CSAS^21/21^*  *C155>CSAS CSAS^Mi/21^*  *Repo>CSAS CSAS^Mi/21^*  *Repo>_ CSAS^21/21^*  *Gli>_ CSAS^21/21^*  *Mz709>_ CSAS^21/21^*  *C155>_ CSAS^21/21^*  *Mj85b>_ CSAS^21/21^*  *1407>_ CSAS^21/21^*  *_>CSAS CSAS^21/21^*  *R85G01>_ CSAS^21/21^*  *R56F03>_ CSAS^21/21^*  *dEAAT1>_ CSAS^21/21^*  *C164>CSAS CSAS^21/21^*  *^^^_>CSAS-RNAi*  *^^^C155>CSAS-RNAi*  *^^^Repo>_*  *^^^Repo>CSAS-RNAi*  *C155>CSAS-FLAG*  *C155>_*  *_>CSAS-FLAG*  *^#^_>DSiaT-RNAi*  *^#^C155>_*  *^#^Repo>_*  *^#^Repo>DSiaT-RNAi*  *^#^C155>DSiaT-RNAi*  *DSiaT^S23/S23^*  *C155>NANS CSAS^Mi/21^*  *_>CSAS+NANS CSAS^Mi/21^*  *C155>CSAS+NANS CSAS^Mi/21^*  *CSAS^21/+^*  *Act>_*  *Act> CSAS[5]*  *Act> CSAS[15]*  *_> CSAS[15*  *^&^WT*  *^&^CSAS^21/21^*  *Repo>CSAS*  *^§^C155>_*  *^§^C155>_ DSiaT^L22/L22^*  *^§^C155>DSiaT DSiaT^L22/L22^*  *DSiaT^L22/L22^*  *^$^WT*  *^$^DSiaT^L22/L22^*  *^$^DSiaT^L22/+^* | *w^1118^ Canton S*  *w^1118^ P{GawB}elav^C155^*  *w^1118^; P{w[+m*]=GAL4}repo/TM3, Sb^1^*  *w^1118^; P{w[+mW.hs]=GawB}insc[Mz1407]*  *w^1118^; P{w[+mC]=Act5CGAL4}17bFO1/TM6B,Tb^1^*  *w^1118^; P{w[+mW.hs]=GawB}C164*  *w^1118^; P{w[+mC]=AyGAL4}25/CyO*  *w^1118^; P{y[+t7.7] v[+t1.8]=TRiP.HMC02922}attP40*  *w^1118^; P{y[+t7.7] w[+mC]=13XLexAop2-mCD8::GFP}attP2*  *w^1118^; P{w[+mC]=lexAop-2xhrGFP.nls}2a*  *w^1118^; P{y[+t7.7] w[+mC]=8XLexAop2-FLPL}attP40*  *w*; P{w[+mW.hs]=GawB}elav[C155], P{w[+mC]=UAS-mCD8::GFP.L}*  *w^1118^; P{y[+t7.7] w[+mC]=GMR85G01-GAL4}attP2*  *w^1118^; P{y[+t7.7] w[+mC]=GMR56F03-GAL4}attP2*  *w^1118^; P{attP,y+,w3'}VIE-260B*  *w*; P{w[+mC]=Eaat1-GAL4.R}2*  *w^1118^; Gli-Gal4/ CyO*  *w^1118^; MZ709-Gal4*  *w^1118^ Mj85b-Gal4*  *w^1118^; UAS-CSAS*  *w^1118^; UAS-CSAS-FLAG*  *w^1118^; UAS-DSiaT*  *y^1^ w* Mi{PT-GFSTF.0}para^MI08578-GFSTF.0^*  *w^1118^; CSAS^21^*  *w^1118^; CSAS^Mi^*  *w^1118^; DSiaT^L22^*  *w^1118^; DSiaT^S23^*  *w^1118^; PBac{CH322-158A02}VK00027*  *w^1118^; PBac{BAC-CSAS-LexA}VK00027*  *w^1118^; BAC-DSiaT-HA*  *w^1118^; UAS-NANS*  *_ _*  *w^1118^; BAC-CSAS-LexA/+; P{y[+t7.7] w[+mC]=13XLexAop2-mCD8::GFP}attP2/+*  *w^1118^; BAC-CSAS-LexA P{w[+mC]=lexAop-2xhrGFP.nls}2a /+*  *w^1118^; CSAS^21^/ CSAS^21^ PBac{CH322-158A02}VK00027*  *w^1118^; + /P{y[+t7.7] w[+mC]=8XLexAop2-FLPL}attP40 ; CSAS^Mi^ PBac{BAC-CSAS-LexA}VK00027 / UAS-CSAS CSAS^Mi^*  *w^1118^; P{w[+mC]=AyGAL4}25 / + ; CSAS^Mi^ PBac{BAC-CSAS-LexA}VK00027 / UAS-CSAS CSAS^Mi^*  *w^1118^; P{w[+mC]=AyGAL4}25 / P{y[+t7.7] w[+mC]=8XLexAop2-FLPL}attP40; CSAS^Mi^ PBac{BAC-CSAS-LexA}VK00027 / UAS-CSAS CSAS^Mi^*  *w^1118^; CSAS^Mi^/ CSAS^21^*  *w^1118^; CSAS^21^/ CSAS^21^*  *w^1118^; P{w[+m*]=GAL4}repo CSAS^21^/ UAS-CSAS CSAS^21^*  *w^1118^; Gli-Gal4/+; CSAS^21^/ UAS-CSAS CSAS^21^*  *w^1118^; Mz709-Gal4 CSAS^Mi^/ UAS-CSAS CSAS^21^*  *w^1118^; P{y[+t7.7] w[+mC]=GMR85G01-GAL4}attP2 CSAS^Mi^/ UAS-CSAS CSAS^21^*  *w^1118^; P{y[+t7.7] w[+mC]=GMR56R03-GAL4}attP2 CSAS^Mi^/ UAS-CSAS CSAS^21^*  *w^1118^; P{w[+mC]=Eaat1-GAL4.R}2/+; CSAS^Mi^/ UAS-CSAS CSAS^21^*  *w^1118^ P{GawB}elav^C155^/ w^1118^ ; CSAS^21^/ UAS-CSAS CSAS^21^*  *w^1118^ Mj85b/ w^1118^ ; CSAS^21^/ UAS-CSAS CSAS^21^w^1118^; P{w[+mW.hs]=GawB}insc[Mz1407]/+; CSAS^21^/ UAS-CSAS CSAS^21^*  *w^1118^; UAS-CSAS CSAS^21^/ CSAS^Mi^*  *w^1118^ P{GawB}elav^C155^/ w^1118^ ; CSAS^Mi^/ CSAS^21^*  *w^1118^; P{w[+m*]=GAL4}repo CSAS^Mi^/ CSAS^21^*  *w^1118^ P{GawB}elav^C155^/ w^1118^ ; CSAS^Mi^/ UAS-CSAS CSAS^21^*  *w^1118^; P{w[+m*]=GAL4}repo CSAS^Mi^/ UAS-CSAS CSAS^21^*  *w^1118^; P{w[+m*]=GAL4}repo CSAS^21^/ CSAS^21^*  *w^1118^; Gli-Gal4/+; CSAS^21^/ CSAS^21^*  *w^1118^; Mz709-Gal4 CSAS^21^/ CSAS^21^*  *w^1118^ P{GawB}elav^C155^/ w^1118^ ; CSAS^21^/ CSAS^21^*  *w^1118^ Mj85b/ w^1118^ ; CSAS^21^/ CSAS^21^*  *w^1118^; P{w[+mW.hs]=GawB}insc[Mz1407]/+; CSAS^21^/ CSAS^21^*  *w^1118^; UAS-CSAS CSAS^21^/ CSAS^21^*  *w^1118^; P{y[+t7.7] w[+mC]=GMR85G01-GAL4}attP2 CSAS^Mi^/ CSAS^21^*  *w^1118^; P{y[+t7.7] w[+mC]=GMR56F03-GAL4}attP2 CSAS^Mi^/ CSAS^21^*  *w^1118^; P{w[+mC]=Eaat1-GAL4.R}2/+; CSAS^Mi^/ CSAS^21^*  *w^1118^; P{w[+mW.hs]=GawB}C164/+; CSAS^21^/ UAS-CSAS CSAS^21^*  *w^1118^ UAS-dcr2/+; P{attP,y+,w3'}VIE-260B / +; CSAS^21^/ +*  *w^1118^ P{GawB}elav^C155^/ w^1118^ UAS-dcr2; P{attP,y+,w3'}VIE-260B /+; CSAS^21^/+*  *w^1118^ UAS-dcr2 / w^1118^; P{w[+m*]=GAL4}repo CSAS^21^/ +*  *w^1118^ UAS-dcr2 / w^1118^; P{attP,y+,w3'}VIE-260B / + ;*  *P{w[+m*]=GAL4}repo CSAS^21^/ +*  *w^1118^ P{GawB}elav^C155^/ w^1118^ ; UAS-CSAS-FLAG/ +*  *w^1118^ P{GawB}elav^C155^/ w^1118^*  *w^1118^; UAS-CSAS-FLAG/ +*  *w^1118^; P{y[+t7.7] v[+t1.8]=TRiP.HMC02922}attP40 / +; DSiaT^S23^/ +*  *w^1118^ P{GawB}elav^C155^/ w^1118^ ; DSiaT^S23^/ +*  *w^1118^ ; DSiaT^S23^/+; P{w[+m*]=GAL4}repo / +*  *w^1118^; P{y[+t7.7] v[+t1.8]=TRiP.HMC02922}attP40 / DSiaT^S23^; P{w[+m*]=GAL4}repo / +*  *w^1118^ P{GawB}elav^C155^ / w^1118^;*  *P{y[+t7.7] v[+t1.8]=TRiP.HMC02922}attP40 / DSiaT^S23^*  *w^1118^; DSiaT^S23^/ DSiaT^S23^*  *w^1118^ P{GawB}elav^C155^/ w^1118^ ; CSAS^Mi^/ UAS-NANS CSAS^21^*  *w^1118^; UAS-CSAS CSAS^Mi^/ UAS-NANS CSAS^21^*  *w^1118^ P{GawB}elav^C155^/ w^1118^ ; UAS-CSAS CSAS^Mi^/ UAS-NANS CSAS^21^*  *w^1118^; CSAS^21^/ +*  *w^1118^; P{w[+mC]=Act5CGAL4}17bFO1 / +*  *w^1118^; UAS-CSAS[5] / +; P{w[+mC]=Act5CGAL4}17bFO1 / +*  *w^1118^; P{w[+mC]=Act5CGAL4}17bFO1 / UAS-CSAS[15]*  *w^1118^; UAS-CSAS[15] / +*  *w^1118^; P{w[+m*]=GAL4}repo / +*  *w^1118^; P{w[+m*]=GAL4}repo CSAS^21^/ CSAS^21^*  *w^1118^; P{w[+m*]=GAL4}repo / UAS-CSAS*  *y^1^ w* Mi{PT-GFSTF.0}para^MI08578-GFSTF.0^ / w^1118^ P{GawB}elav^C155^*  *y^1^ w* Mi{PT-GFSTF.0}para^MI08578-GFSTF.0^ / w^1118^ P{GawB}elav^C155^; DSiaT^L22^/ DSiaT^L22^*  *y^1^ w* Mi{PT-GFSTF.0}para^MI08578-GFSTF.0^ / w^1118^ P{GawB}elav^C155^;*  *DSiaT^L22^/ DSiaT^L22^; UAS-DSiaT / +*  *w^1118^; DSiaT^L22^/ DSiaT^L22^*  *w* P{w[+mW.hs]=GawB}elav[C155], P{w[+mC]=UAS-mCD8::GFP.L} / +*  *w* P{w[+mW.hs]=GawB}elav[C155], P{w[+mC]=UAS-mCD8::GFP.L} / + ; DSiaT^L22^/ DSiaT^L22^*  *w* P{w[+mW.hs]=GawB}elav[C155], P{w[+mC]=UAS-mCD8::GFP.L} / + ; DSiaT^L22^/ +* | (Dubnau et al., 2001)  BDSC 458  BDSC 7415  BDSC 8751  BDSC 3954  BDSC 33807  BDSC 3953  BDSC 44528  BDSC 32203  BDSC 29954  BDSC 55820  BDSC 5146  BDSC 40436  BDSC 39157  VDRC 101396  BDSC 8849  (Auld et al., 1995; Ito et al., 1995)  (Dubnau et al., 2001)  (Islam et al., 2013)  (Islam et al., 2013)  (Repnikova et al., 2010)  (Ravenscroft et al., 2020)  (Islam et al., 2013)  (Islam et al., 2013)  (Repnikova et al., 2010)  This study  This study  This study  This study  _ _  Fig. 2  Fig. 2  Fig. 2  Fig. 2  Fig. 2  Fig. 2  Fig. 2, 3, 5-8  Fig. 2, 3, 5-8  Fig. 3, 5, 8  Fig. 3  Fig. 3  Fig. 3  Fig. 3  Fig. 3  Fig. 3  Fig. 3  Fig. 3  Fig. 3  Fig. 3  Fig. 3, 6  Fig. 3  Fig. 3  Fig. 3  Fig. 3  Fig. 3  Fig. 3  Fig. 3  Fig. 3  Fig. 3  Fig. 3  Fig. 3  Fig. 3  Fig. 3  Fig. 3    Fig. 3  Fig. 3  Fig. 3  Fig. 3  Fig. 3  Fig. 4  Fig. 4  Fig. 4  Fig. 4  Fig. 4  Fig. 4  Fig. 6  Fig. 6  Fig. 6  Fig. 7  Fig. 7  Fig. 7  Fig. 7  Fig. 7  Fig. 8  Fig. 8  Fig. 8  Fig. 9  Fig. 9  Fig. 9  Fig. 9  Fig. 9  Fig. 9  Fig. 9 |
